# Murine Leukemia Viruses: Objects and Organisms

**DOI:** 10.1155/2011/403419

**Published:** 2011-11-15

**Authors:** Alan Rein

**Affiliations:** HIV Drug Resistance Program, National Cancer Institute-Frederick, Frederick, MD 21702, USA

## Abstract

Murine leukemia viruses (MLVs) are among the simplest retroviruses. Prototypical gammaretroviruses encode only the three polyproteins that will be used in the assembly of progeny virus particles. These are the Gag polyprotein, which is the structural protein of a retrovirus particle, the Pol protein, comprising the three retroviral enzymes—protease, which catalyzes the maturation of the particle, reverse transcriptase, which copies the viral RNA into DNA upon infection of a new host cell, and integrase, which inserts the DNA into the chromosomal DNA of the host cell, and the Env polyprotein, which induces the fusion of the viral membrane with that of the new host cell, initiating infection. In general, a productive MLV infection has no obvious effect upon host cells. Although gammaretroviral structure and replication follow the same broad outlines as those of other retroviruses, we point out a number of significant differences between different retroviral genera.

## 1. Introduction

A virus can be viewed as a rather regular, relatively simple physical object. Alternatively, it can be seen as a living organism, evolving in response to selective pressures. Both views are correct! This paper will outline very briefly some of the characteristics of murine leukemia viruses (MLVs), keeping both views in mind. We will try to point out the distinctive features of these retroviruses, which are often taken as prototypes of the gammaretrovirus genus. (Retroviruses include Spumaretroviruses (also known as “foamy viruses”) and Orthoretroviruses; the latter are divided into six genera, that is, alpha-, beta-, gamma-, delta-, epsilon-, and lenti-retroviruses [1].)

MLVs have been studied for many years, beginning in the 1950s, when it was realized that leukemia could be transmitted to newborn mice by a filterable agent [2–4]. They have provided many insights into the general phenomenon of leukemogenesis. The MLV genome has also been used as the starting material in the development of vectors for gene therapy. Finally, MLVs have often been viewed as “model” retroviruses. In fact, while they have been very useful in answering questions about retroviruses and their hosts, there are many ways in which gammaretroviruses differ from other retroviruses: it should never be assumed that a given property of one genus will hold for another.

The best-studied retrovirus is, of course, human immunodeficiency virus (HIV-1), which is a lentivirus. One striking contrast between MLVs and HIV-1 is the relative simplicity of MLVs. As discussed below, MLVs only encode the proteins that will be assembled into the progeny virus particles, whereas HIV-1 encodes six additional so-called “accessory” proteins. Indeed, because of this distinction, HIV-1 has frequently been called a “complex” retrovirus, in contrast to the “simple” retroviruses such as MLV, the proper objects of study of simple retrovirologists. 

The two viruses also differ in that HIV-1 can efficiently infect nondividing cells, while MLV generally does not [5, 6] (but see also [7, 8]). The ability of HIV-1 to infect nondividing cells is a critical element in its pathogenicity. 

Yet another cardinal difference between MLVs and HIV-1 is that HIV-1-infected cells usually die rapidly (within a few days at most) after infection. In contrast, at the cellular level MLV infection seems almost completely benign: in general, there are no detectable effects of productive MLV infection upon the growth, physiology, or morphology of the cells. HIV-1 viremia is maintained in infected people by continual infection of new cells, replacing the cells killed by infection. We do not know how much infection is occurring in an MLV-infected, viremic mouse, but since the virus does not generally kill its host cells, the rate of new infections may be far lower than with HIV-1. It should be noted that the drugs used in highly active antiretroviral therapy of HIV-infected people act by blocking new infections; thus, it is possible that analogous therapies would have only minimal effects on MLV viremia.

## 2. MLV: The Physical Object

### 2.1. MLV Virions

**Figure 1:**
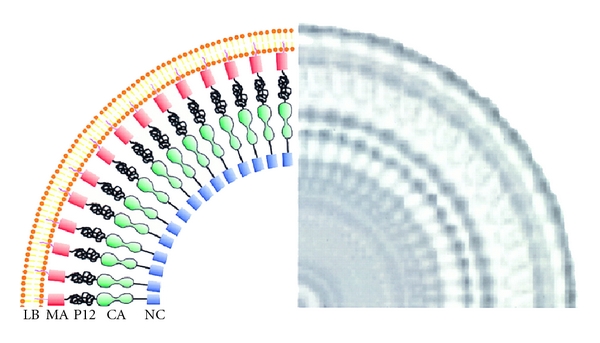
Structure of an immature MLV particle. A segment from a rotationally averaged cryoelectron microscopic image of a single immature MLV particle is shown on the right. As indicated on the left, the particle is bounded by a lipid bilayer (“LB”), and the MA domain of Gag (pink) is associated with the inner leaflet of the bilayer. Interior to the MA domain is a zone of low density, presumably corresponding to the p12 domain. The most conspicuous feature of the image is the “railroad tracks”, representing the two domains within the CA domain (green), followed by the NC domain (blue) with bound RNA. The Pol and Env proteins are not visible in this image. As the particle is ~100 nm in diameter and the Gag molecules are ~20 nm in length, there is a region ~60 nm in diameter largely occupied by solvent in the center of the particle. (Reproduced from [9]. Copyright 1998, National Academy of Sciences, USA.)

The overall structures of virus particles are probably very similar for all Orthoretroviridae. The virus is pleomorphic, but roughly spherical, with a diameter of ~100–120 nm [9]. It is released from the cell as an “immature particle”, in which several thousand rod-shaped Gag polyprotein molecules are arranged, in an incomplete or imperfect hexameric lattice, as radii of the sphere (see Figure 1). The sphere is bounded by a lipid bilayer derived from the plasma membrane of the virus-producing cell. The N-terminal matrix (MA) domains of the Gag molecules are in contact with the lipid bilayer and their C-terminal nucleocapsid (NC) domains project into the interior of the particle, presumably in contact with RNA. They are approximately 20 nm long and only 2-3 nm in diameter. The particle also contains ~1–300 Gag-Pol polyprotein molecules, in which Gag is extended at its C-terminus by protease (PR), reverse transcriptase (RT), and integrase (IN). Finally, trimers of the envelope (Env) polyprotein span the membrane, with the gp70 surface glycoprotein (SU) on the exterior of the particle, complexed with the p15E transmembrane (TM) protein. Roughly 2.5 × 10^4^ nucleotides' worth of RNA, representing only a few per cent of the mass of the particle, are also present in the virion. Some cellular proteins are also packaged: this has been documented in great detail in HIV-1 [10] but is also true in MLV [11].

After the particle is released from the cell, it undergoes maturation. PR cleaves Gag into four cleavage products, that is, MA, p12, capsid (CA), and NC. The Pol moiety of Gag-Pol is also cleaved to release free PR, RT, and IN proteins, and the C-terminal 16 residues of TM (the “R peptide”) are removed, producing the mature TM protein p15E (in some papers, this shorter species is called p12E; the longer precursor has been called either p15E or Pr15E). The cleavages in Gag cause a major change in the overall architecture of the virion, with CA molecules reassembling in the interior of the particle into a polygonal structure, the “mature core” of the particle. This new structure encloses a complex of the viral RNA with NC protein; RT and IN are also presumed to be within this structure.

### 2.2. The MLV Replication Cycle

**Figure 2:**
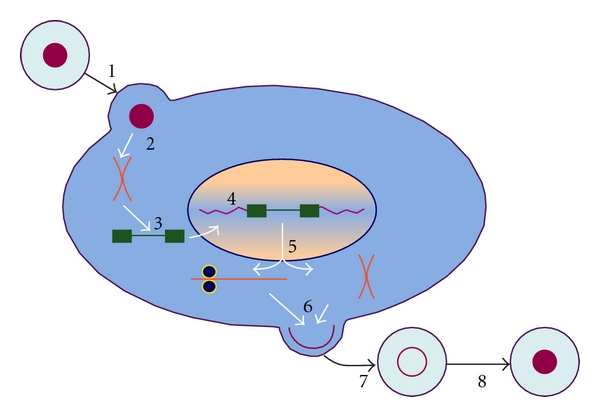
The orthoretroviral replication cycle. Infection is initiated when the mature, infectious virion binds to a receptor on the surface of the new host cell. The Env protein of the virus induces fusion between the viral membrane and the cell membrane (Step 1). Within the cytoplasm, the mature core dissociates (Step 2) and the dimeric viral RNA (shown in orange) is copied (Step 3) into double-stranded DNA (shown in green). The DNA copy enters the nucleus (probably when the nuclear membrane breaks down during mitosis) and is inserted into the chromosomal DNA of the cell (Step 4). The DNA is transcribed and the RNA product is exported from the nucleus (Step 5); within the cytoplasm, some molecules will be translated into viral proteins, and others are destined for encapsidation into progeny virus particles. The viral components assemble into budding virions (Step 6), which are released from the cell as immature particles (Step 7). Finally, PR cleaves the viral proteins, converting immature particles into mature, infectious virions (Step 8). It is possible that DNA synthesis actually occurs within the mature core rather than after dissociation of the core as shown here.

As with all orthoretroviruses, infection is initiated by the binding of the SU glycoprotein on the exterior of the mature, infectious virion to a receptor on the surface of the new host cell (see Figure 2). This binding event triggers dramatic changes in Env, leading to the release of the SU component and conformational rearrangement of TM. The ultimate result is the fusion of the viral membrane with the plasma membrane.

 The fusion of the two membranes leads to the deposition of the contents of the virion in the cytoplasm of the cell. Once in the cytoplasm, the viral RNA is copied by the RT into a single molecule of dsDNA. This DNA is somehow conveyed into the nucleus, where the IN protein catalyzes its insertion into chromosomal DNA. 

Once the viral DNA is integrated into host DNA, it is termed the “provirus”. It is transcribed and translated by normal host-cell machinery. The encoded proteins are trafficked to the plasma membrane, where they assemble into progeny virus particles. The immature particles are released from the cell with the help of the cellular “ESCRT” machinery [23] and subsequently undergo maturation as the PR in the virus cleaves the viral polyproteins. The particle is not capable of initiating a new infection until maturation has taken place.

### 2.3. The MLV Genome

**Figure 3:**
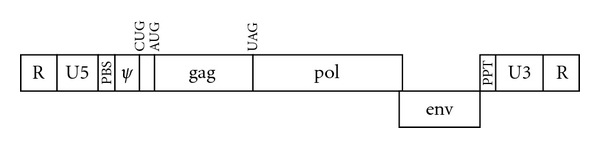
The MLV genome. The viral RNA in Moloney MLV is 8332 nt in length [18]. “R” sequence, 68 nt in length, is identical at both ends of the RNA. The 5′ copy of R is followed by U5 sequences and then by the PBS (nt 146–163). The long 5′ untranslated region in the RNA also includes the *ψ* packaging signal; contained within this signal in some MLV isolates is the CUG codon at which glyco-Gag translation is initiated (nt 357). The initiation codon for the “normal” Gag protein is at nt 621. The *gag* and *pol* coding sequences are in the same frame; they are separated by a UAG termination codon, which in turn is followed immediately by a 57-base signal, including an RNA pseudoknot, inducing the inefficient translation of the UAG as glutamine. The Env protein is translated from a spliced mRNA. The polypurine tract (PPT, nt 7803–7815) is the primer for +-strand DNA synthesis and is followed by the U3 and R regions. U3 (nt 7816–8264) is placed at the 5′ end of the DNA copy of the genome synthesized during infection; it contains promoter and enhancer sequences governing the initiation of transcription at the beginning of R.

The RNA genome of MLV can be divided into coding and noncoding regions and is shown schematically in Figure 3.

#### 2.3.1. Coding Regions

The only proteins encoded by the MLV genome are the three polyproteins that will make up the progeny virus particles: Gag, the structural protein of the immature virus particle, Pol, comprising the PR, RT, and IN enzymes, and Env, the SU and TM proteins that jointly mediate the entry of an infectious virus particle into a new host cell to initiate infection [18]. In some MLV isolates, an alternative form of Gag, with an N-terminal extension, is also synthesized; this “glyco-Gag” is discussed below.

As in all orthoretroviruses, the three coding regions are arranged, from 5′ to 3′, Gag : Pol : Env. The Pol proteins are initially synthesized together with Gag, in a large Gag-Pol fusion polyprotein. Gag and Gag-Pol are both translated from full-length viral RNA, identical in sequence to the genomic RNA present in the virion. It seems likely that the Gag-Pol polyprotein is incorporated into assembling virions due to “coassembly” of its Gag moiety with Gag polyprotein molecules. Successful replication of the virus requires maintenance of an optimal ratio (on the order of 20 : 1) between the Gag and Gag-Pol proteins; indeed, no detectable virus particles are formed in cells expressing only Gag-Pol [24]. This may be because Gag-Pol is more than 3 times the mass of Gag, and thus, there may not be space within the particles for very many Pol domains. This optimal ratio is achieved by finely tuned translational suppression of the termination codon at the end of the Gag coding region. 

Remarkably, different retroviruses use fundamentally different mechanisms of translational suppression. In the gammaretroviruses such as MLV (and epsilonretroviruses, a genus about which very little is known), Gag and Pol are in the same reading frame, separated by a single termination codon. MLV RNA contains a 57-base cis-acting signal immediately 3′ of the termination codon [25]. This signal induces the insertion of glutamine (normally encoded by CAG), rather than termination, in response to the UAG termination codon in about 5% of the translation products; the resulting product is extended by translation of the entire Pol coding region [26]. Similar results are obtained when the UAG is replaced by UGA or UAA [27, 28]. Thus, these viruses operate in essence by “mis-translation” of the termination codon as a sense codon. In contrast, in all other genera, the suppression occurs before the ribosomes encounter the termination codon and is completely independent of this codon. In these viruses, Pol is encoded in the “−1” frame relative to Gag. A signal in these viral RNAs before the end of the Gag coding region induces a fraction of the ribosomes to advance two, rather than three bases at a specific codon so that translation by this subset of ribosomes is shifted from the Gag frame to the Pol frame [29, 30]. (In some retroviruses, there are two frameshifting events, one extending Gag to produce Gag-PR and the second extending Gag-PR to yield Gag-PR-RT-IN.) A detailed discussion of translational suppression in retroviruses may be found in Hatfield et al. [31]. 

The Env protein of MLV, like that of other orthoretroviruses, is translated from a singly spliced mRNA. There is an overlap of 58 bases between the end of the Pol coding region and the beginning of the Env coding region.

#### 2.3.2. Noncoding Regions

Like the RNAs of all orthoretroviruses, MLV RNA also contains a set of cis-acting signals that are essential for its function as a viral genome. These include the “primer binding site” (PBS), the polypurine tract (PPT), the “packaging signal” or *ψ*, sequences required for insertion, by IN, of the DNA form of the viral genome into cellular DNA, and the promoter and enhancer sequences within the LTR.

The PBS is an 18-base stretch that is complementary to the last 18 bases of a cellular tRNA molecule. In MLVs, this is usually tRNA^Pro^, but MLVs using tRNA^Gln^ have also been found. Within the virion, the tRNA is hybridized to the viral RNA; when the virus enters a new host cell, the tRNA serves as the primer for reverse transcription. The PBS is located ~145 bases from the 5′ end of the RNA and ~460 bases 5′ of the beginning of the Gag coding region. The first deoxynucleotide to be added to the tRNA during reverse transcription is determined by pairing with the base immediately 5′ of the PBS, and this base is the 5′ terminus of the first (minus) strand in the final DNA product. In other words, this site is the “right” end of the final double-stranded DNA product of reverse transcription.

In general, during reverse transcription the RNA is copied by the polymerase activity of RT and is progressively degraded, shortly after being copied, by the RNase H activity of RT. However, an exceptional stretch of ~15 purines near the 3′ end of retroviral RNAs (the PPT) is specifically resistant to this degradation. Having survived reverse transcription, this fragment of the viral RNA is the primer for synthesis of the second (plus) strand of DNA. The base immediately 3′ of the PPT encodes the first base of the plus strand of the DNA copy, that is, the 5′ end of the plus strand or “left” end of the double-stranded DNA.

These sequences at the two ends of the final DNA product are, of course, the sequences joined by IN to host-cell chromosomal DNA during the integration reaction. The two ends form an inverted repeat (reviewed in [32]). In Moloney MLV, the sequence of the “plus” strand at the right edge is 5′ GGGGTCTTTCA 3′, while that at the left edge is 5′ TGAAAGACCCC 3′. The bases at the 3′ ends of the plus strand on the right edge, and the 5′ end of the left edge, are joined to cellular DNA, but it is the internal bases in these sequences that are essential for IN recognition [33, 34].

All orthoretroviral genomic RNAs are, as noted above, mRNAs. They resemble cellular mRNAs in having a 5′ cap and 3′ poly (A) tail. In fact, under certain conditions, retrovirus particles can encapsidate cellular mRNAs [35]. Thus, the viral RNAs are evidently in competition with cellular mRNAs for incorporation into the virions. Intact retroviral RNAs are selectively incorporated because they contain a “packaging signal”, giving them an advantage in this competition. 

**Figure 4:**
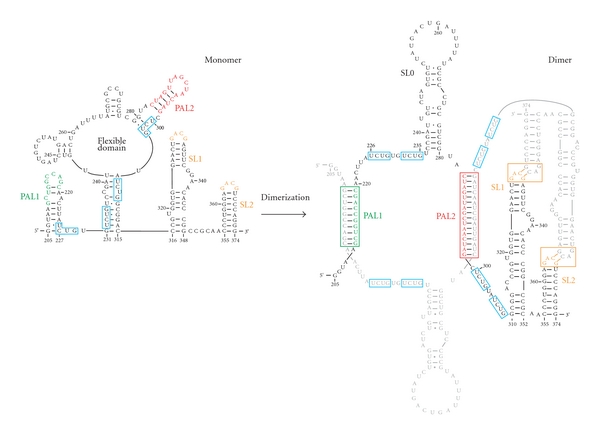
The Moloney MLV dimerization/packaging signal. The figure shows the secondary structure of the 170-base “minimal dimerization active sequence” (nt 205–374) [19] in both monomeric and dimeric forms. Two palindromic sequences, “PAL1” (green) and “PAL2” (red), are contained within stem loops in the monomer but open out and pair intermolecularly in the dimer. The two monomers are also connected in the dimer by base pairing between the “CG” moieties in the “GACG” loops of a pair of stem loops (“SL1” and “SL2”, orange). The RNA also contains two motifs with the sequence UCUG-UPu-UCUG (blue boxes); these are partially or fully base-paired in the monomer but become unpaired as a result of the RNA rearrangements accompanying the intermolecular base pairing of PAL1 and PAL2. These bases are a crucial element in *ψ*, as replacement of the four UCUG sequences with UCUA prevents selective packaging of the viral RNA; the exposure of these bases in dimers, but not monomers, presumably explains the selective packaging of dimeric RNA [20]. (Figure reproduced from Trends in Biochemical Sciences, Copyright 2011, with permission from Elsevier [21].)

Recent structural studies have shed considerable light on the nature of the packaging signal in Moloney MLV RNA (see Figure 4) [20, 36]. Briefly, in all orthoretroviruses, the viral RNA is actually packaged in dimeric form, with two molecules of the viral RNA linked by a limited number of intermolecular base pairs. The primary location of these base pairs is in the “leader”, between the PBS and the beginning of the Gag coding sequence. MLV RNA, like that of all gammaretroviruses, contains a pair of stem loops in this region with the sequence GACG in the loop [37]. Both NMR and chemical-probing data show that when MLV RNA dimerizes, the “CG” within each of these GACG's pairs with the CG in the other monomer (note that “CG” is a 2-base palindrome, the shortest possible palindromic sequence) [38, 39]. Further, two other stem loops in the monomers open out and pair intermolecularly. Most interestingly, this change entails a shift in register so that some of the bases which are paired in intramolecular structures in the monomers become unpaired in the dimers. These bases include two copies of the motif UCUG-UPu-UCUG. Several kinds of experiments [20] show that this motif is essential for high-affinity binding by recombinant MLV Gag protein, that these bases are occupied by NC protein within mature MLV particles, and that they are crucial to selective packaging. These results explain why dimers, but not monomers, of viral RNA are selectively packaged and also establish that the specific, high-affinity binding of Gag to *ψ* is responsible for selective packaging. 

During reverse transcription, sequences from near the 3′ end of the viral RNA (“U3” sequences) are placed at the 5′ end, as well as near the 3′ end, of the viral DNA. (Conversely, U5 sequences, from near the 5′ end of the RNA, are placed at the 3′ end as well as near the 5′ end of the DNA.) Following integration of the viral DNA, the U3 sequences at the 5′ end constitute the promoter and enhancers driving the transcription, by Pol II, of the integrated DNA. U3 sequences include a dense collection of transcription factor-binding sites; they were used in the experiments that originally demonstrated the existence of enhancers [40] and play a major role in determining the tissue tropism and pathogenicity of the virus (reviewed in [41]). The placement of the U3 sequences, which are internal in viral RNA, upstream of the transcriptional start site in the DNA is an elegant solution to the problem of how to ensure that the viral sequences will lie 3′ of a promoter, as required for Pol II transcription.

### 2.4. MLV Proteins

#### 2.4.1. Gag

In essence, the orthoretrovirus particle is constructed by assembly of Gag protein molecules. All orthoretroviral Gag proteins contain at least three domains, which will give rise to three distinct proteins in the mature virus. The MA domain at the N-terminus of Gag is responsible for targeting the protein to the plasma membrane of the virus-producing cell. In MLV, as in most retroviruses, the N-terminus of Gag is modified by the 14-carbon saturated fatty acid, myristic acid [42]; this modification is important for the plasma-membrane association of Gag [43]. The CA domain is the locus of most, if not all, of the interactions between Gag molecules leading to the assembly of the immature virion. After the CA molecules are released from the Gag polyprotein by PR, they reassemble into the mature core. The NC domain plays a predominant role in the interactions of Gag proteins with RNAs, and free NC protein is an essential cofactor in reverse transcription during infection. In general, there is considerable structural conservation between the Gag proteins in different orthoretroviral genera, despite the almost complete lack of conservation of primary sequences.

**Figure 5:**
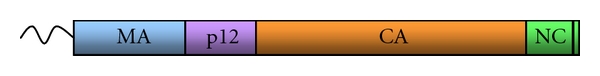
MLV Gag protein. The MLV Gag protein is modified at its N-terminus by the 14-carbon fatty acid myristic acid. It is cleaved during virus maturation into MA, p12, CA, and NC; most of the NC molecules are also cleaved 4 residues before their C-terminus.

MLV Gag differs in two important respects from the canonical MA-CA-NC Gag structure (see Figure 5). First, it contains an additional domain, called p12, situated between MA and CA. p12 contains the Pro-Pro-Pro-Tyr “late domain” of MLV [44]; this motif interacts with an Nedd4-like ubiquitin ligase to promote the release of the assembled virion from the host cell [45]. p12 also participates in the infection process, but these additional functions are not well understood. It is part of the “preintegration complex”, a collection of proteins from the infecting virus particle that accompany the newly synthesized viral DNA into the cell nucleus [46], and some mutations in p12 interfere with proper integration [47, 48]. Surprisingly, there are regions within p12 in which sequence changes seem to have no major effect on viral function [49, 50], and the maturation cleavage between MA and p12, unlike the other cleavages, is not absolutely essential for viral infectivity [51]. It is extremely proline-rich (18 of its 84 residues (21%) are prolines), and it has been described as “unstructured” on the basis of NMR data [52]. However, recombinant MLV Gag protein is an extended rod in solution, and the prolines in the p12 domain contribute to its rigidity (Datta et al., manuscript in preparation). It seems likely that this domain in Gag can assume any of a number of rigid conformations containing short polyproline helices.

Second, some, but not all, MLV isolates encode an alternative form of the Gag polyprotein, called “glyco-Gag” or gPr80^Gag^. This protein differs in sequence from “standard” Gag in that it is extended N-terminally. Synthesis of glyco-Gag is initiated at a CUG codon in a favorable context for translation initiation, 264 bases 5′ of the normal Gag AUG initiation codon [53]. The N-terminal extension includes a signal sequence so that this protein (unlike standard Gag) is synthesized in the rough endoplasmic reticulum and processed in the Golgi apparatus. Relatively little glyco-Gag is incorporated into virions [54]. Because of a sequence polymorphism at the site of the CUG initiator, XMRV does not encode glyco-Gag. 

The functional significance of glyco-Gag is still not clear. Early studies showed that it is not essential for replication of MLV in cell culture, but is needed for efficient replication and pathogenicity in mice [55, 56]. It was recently reported that the correct assembly of standard MLV Gag into spherical immature particles in cell cultures is impaired in the absence of glyco-Gag [57]; new data indicates that the presence of glyco-Gag directs virion assembly to lipid rafts and that this function involves the cellular La protein [58]. Remarkably, glyco-Gag can also complement Nef deletions in HIV-1 [59].

MLV Gag is also unusual among orthoretroviral Gags in that its NC domain only contains a single zinc finger rather than two as in most genera. The zinc-coordinating residues have the spacing C-X_2_-C-X_4_-H-X_4_-C, as in all orthoretroviral NC proteins. This 14-residue motif plays a critical role in the selective packaging of genomic RNA, among other functions [60, 61]. The last 4 residues of NC are removed from the majority of Gag molecules, as they are from Gag-Pol molecules, during virus maturation [26, 62].

#### 2.4.2. Pol

As noted above, the products of cleavage of the Gag-Pol polyprotein include PR, RT, and IN. PR catalyzes the cleavages leading to virus maturation; like all retroviral PRs, it is an aspartic protease which is only active as a dimer [63, 64].

RT synthesizes the DNA copy of the viral genome during infection. This function involves three enzymatic activities: RNA-templated DNA synthesis, DNA-templated DNA synthesis, and degradation of the RNA strand in an RNA:DNA hybrid, eliminating the RNA template immediately after synthesis of the complementary DNA strand. MLV RT is apparently active as a monomeric protein [65, 66] unlike the RT enzymes of alpharetroviruses and lentiretroviruses, which are both heterodimers [67]. 

Retroviral IN enzymes possess two catalytic activities: “3′ end processing”, in which IN removes two nucleotides from the 3′ end of each strand of the DNA to be integrated, and “strand transfer”, in which the new 3′ ends are inserted into chromosomal DNA in the new host cell [32]. MLV IN has not been characterized in detail but is presumed to function as a tetramer [68, 69].

#### 2.4.3. Env

As with all orthoretroviruses, the MLV Env gene product is synthesized in the rough endoplasmic reticulum and glycosylated in the Golgi apparatus. It is also cleaved in the Golgi by a cellular furin-like protease into two fragments, the large, N-terminal surface glycoprotein (gp70^SU^) and the C-terminal transmembrane protein p15E^TM^. A trimer of these heterodimeric SU-TM complexes is then trafficked to the cell surface. As mentioned above, it undergoes an additional cleavage during virus maturation: PR removes the C-terminal 16 residues, also known as the “R peptide”, from the cytoplasmic tail of the TM protein [62, 70]. This maturation cleavage of TM is found in the gammaretroviruses, in Mason-Pfizer monkey virus, a betaretrovirus [71, 72], and in the lentivirus equine infectious anemia virus [73], but not, as far as is known, in other retroviruses.

**Figure 6:**
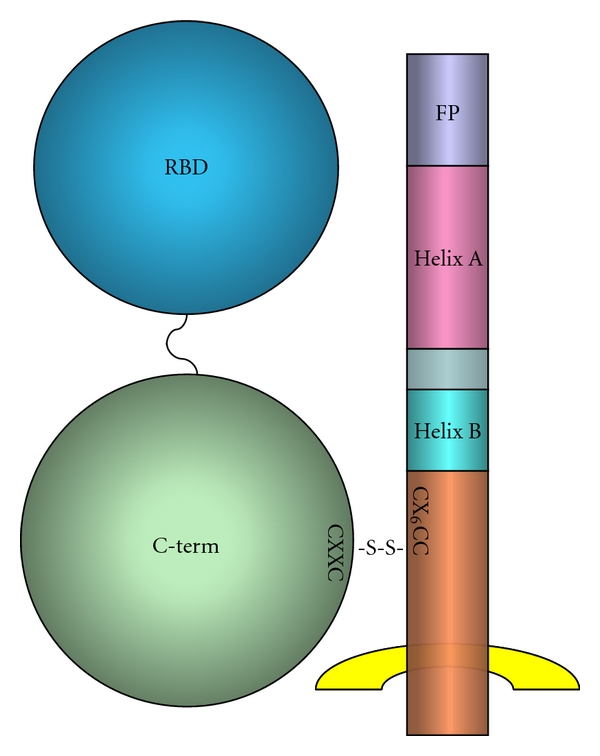
MLV Env protein. MLV Env protein consists of a complex between gp70^SU^ and p15E^TM^. The cartoon shows that gp70 has two domains, RBD at its N-terminus and “C-term” at its C-terminus, separated by a variable, proline-rich linker. P15E contains, from N- to C-terminus, the fusion peptide (FP), an N-terminal helical domain (helix A), a short C-terminal helical domain (helix B), and a C-terminal domain (light pink) which spans the viral membrane (yellow). Gp70 is exclusively external to the virus and is connected to p15E by a disulfide linkage between one of the two cysteines in a CXXC motif within its C-terminal domain and the last cysteine in a CX_6_CC motif in p15E.

MLV Env is depicted schematically in Figure 6. Mature SU of Moloney MLV is 435 residues in length, while TM is 180 residues. In turn, SU contains an N-terminal “receptor-binding domain” (RBD) of ~240 residues, a short, proline-rich “hinge” region, and a highly conserved C-terminal domain [74]. The RBD consists of an antiparallel *β*-sandwich projecting “up” from the surface of the virion, and a highly variable region resting atop this scaffold. Both ends of the RBD contribute to this *β*-sandwich [75]. Sequence alignments and analysis of chimeric SU proteins show that the variable sequences within the RBD make specific contacts with cell-surface receptors. Among the conserved features of SU are a histidine residue near the extreme N-terminus and a CXXC motif in the C-terminal portion of SU. TM protein begins with a very hydrophobic stretch, the “fusion peptide”. A stretch between TM residues 43 and 78 (in Moloney MLV) has a 4-3 repeating pattern of hydrophobic residues that forms a coiled coil. TM also contains a CX_6_CC motif; in the virus particle, there is a disulfide bond joining SU, *via* one of the cysteines in the CXXC, to TM, *via* the last cysteine in the CX_6_CC [76–78]. 

The function of the Env complex is to induce fusion between the membrane surrounding the virus particle and the membrane of a new host cell. As in all orthoretroviruses, the cleavage between SU and TM is absolutely required for Env function [79]. Presumably, this is essential because it places the fusion peptide at the N-terminus of TM rather than in the interior of the Env polyprotein. The removal of the R peptide from the C-terminus of Prp15E during virus maturation is also necessary for the fusogenicity of Env [80, 81]. It seems likely that fusogenic activity would be harmful to the virus-producing cell and that the R peptide is a “safety catch” suppressing this activity until the virus has left the cell. The mechanism by which the R peptide inhibits fusion is not known, but, remarkably, it has the same effect when joined to the influenza HA protein [82]. 

The fusion between the two membranes by the mature Env complex is the end result of an amazing cascade of events. Briefly, binding to the receptor on the plasma membrane induces a conformational change in the RBD. This change is propagated in SU, resulting in the ionization of the one free thiol in its CXXC motif [83]. (The conserved histidine near the N-terminus of SU, which is essential for Env function, may catalyze this ionization [84].) The ionized sulfur then attacks the neighboring cysteine, and the disulfide linkage between SU and TM is replaced by an intra-SU bond between these two cysteines. Breaking the SU-TM bond releases SU from the Env complex, exposing the fusion peptide at the N-terminus of TM. The fusion peptide inserts into the target membrane; this is followed by a major conformational change in TM, in which a C-terminal heptad repeat-like sequence in the TM ectodomain folds against the N-terminal heptad repeat [76]. This shift to a hairpin configuration brings the two membranes into very close apposition; this finally results in the fusion of the two membranes. 

Further studies make it clear that RBD functions not only to bind a receptor on the target cell, but also to prevent the conformational change in TM, leading to membrane fusion, from occurring prematurely, that is, before contact of the virus with the receptor [85, 86]. In fact, under special circumstances infection can occur “in *trans*”, that is, when a *soluble* RBD binds a cell-surface receptor in proximity to the virion [87]. This activity of the MLV Env complex has special consequences for the “MCF” class of MLVs. These “mink cell focus-inducing” or “polytropic” MLVs arise in mice that are viremic for ecotropic MLVs, and are recombinants in which the ecotropic RBD has been replaced by an RBD from an endogenous MLV genome [88–90]. This substitution gives the MCF a different receptor specificity from that of its ecotropic parent [13–15, 91]. The complex of the ecotropic SU protein with the ecotropic receptor on target cells (as in the viremic mice) has been shown to facilitate infection of the cells by MCF virions [92]. 

Remarkably, TM protein performs yet another function for MLV. Immediately proximal to the CX_6_CC motif discussed above is a 20-residue stretch which has potent immunosuppressive activity; this activity is crucial in MLV infections in mice [93, 94]. 

As indicated above, MLVs are polymorphic with respect to their use of cell-surface receptors. In general, when a cell is productively infected with an MLV, the viral Env protein saturates the receptors that it would use for infection, rendering the cell almost completely resistant to superinfection by virus particles that use the same receptor. This resistance makes it possible to group MLV isolates into families sharing common receptors. “Interference” measurements of this kind showed that NIH/3T3 mouse cells have four distinct cell-surface molecules used as receptors by different MLVs, as indicated in Table 1 [91, 95]. This polymorphism is considered in detail in a comprehensive review [96], and is discussed in other articles in this series. It is notable that all receptors used by MLVs contain multiple membrane-spanning domains, unlike the known receptors for most other orthoretroviruses.

## 3. MLV: The Organism

### 3.1. Assays for Infectious MLV

Quantitative virology is virtually impossible without a reliable infectivity assay [97]. Since MLVs generally have no obvious effect on the cells they infect, the opportunities for developing a “plaque” or “focus” assay have been very limited. Two such assays have been devised, each exploiting a specific cell line with a unique response to MLV infection. 

One of these is the “UV-XC” test [98]. XC cells, derived from a rat tumor induced by Rous sarcoma virus, undergo rapid syncytium formation when they come into contact with cells producing ecotropic MLV. This property was used to develop an “indirect” plaque assay: a plate of permissive cells is first infected with the virus, and the virus is allowed to spread in these cells for 5–7 days. At the end of this period, the cells have grown into a confluent monolayer, and the plate contains invisible “foci” of MLV-producing cells. Each focus has arisen by the localized spread of virus from a single cell, infected by a virus in the inoculum, to neighboring cells; several rounds of replication can occur during the assay. This monolayer is then killed by UV-irradiation and overlaid with XC cells. A day later, the XC cells have replaced the original cells; they are fixed and stained, and “plaques”, that is, localized regions of syncytia, are counted. One particular advantage of this assay is that it can be used to measure the infectivity of any ecotropic MLV on any cells; thus, for example, comparing the titer of a single virus preparation on NIH/3T3 cells and Balb/3T3 cells tells one whether the virus is N-tropic, B-tropic, or NB-tropic. On the other hand, the fact that it only detects ecotropic MLVs is a serious limitation of the UV-XC test.

The other quantitative assay for replication-competent MLV is the S+L− assay [99]. S+L− cells are specific cell lines transformed by Moloney sarcoma virus. When these cells are superinfected by an MLV, they become much rounder and more refractile (this may reflect “hypertransformation”, perhaps due to reinfection of the cells with additional copies of Moloney sarcoma virus after it has been rescued by the MLV). In this assay, S+L− cells are infected and allowed to grow for ~5 days; “foci” of rounded cells, which stand out against the confluent monolayer of uninfected S+L− cells, are then scored under a low-power microscope. This assay has the advantage that it will detect any replication-competent MLV, not just members of a specific class. However, it is extremely time consuming. It can also be difficult to distinguish the foci from random irregularities in the cell monolayer, so scoring the assay requires considerable skill and involves some judgment. 

For many, but not all, kinds of experiments, replication-defective “reporter” viruses rescued by MLV can be assayed in lieu of assaying the MLV itself. The reporter viruses originally used in this way were acute transforming viruses; for example, MLVs were grouped into interference families by measuring the ability of Harvey MSV pseudotypes to transform MLV-infected cells [91, 95]. More recently, of course, MLV-derived vectors expressing a variety of genes, such as luciferase, *β*-galactosidase, and green fluorescent protein, have been constructed for use as reporter viruses (e.g., [100]). 

Cell lines have also been developed in which a reporter gene is only expressed following replication in the cell of an MLV. These cells contain an MLV-derived vector which carries a reporter gene in reverse orientation; the reporter gene is interrupted by an intron in the forward orientation. Transcription and splicing yields an RNA in the cell with an uninterrupted, negative-sense copy of the reporter gene; if this RNA is rescued by an MLV, it can be copied into DNA, finally producing an intact reporter gene whose expression can be measured (Aloia et al., manuscript in preparation, but see [101]). This assay has the special advantage that it can be performed by cocultivation of the assay cells with cells producing the virus to be assayed, as well as by infection of the assay cells with cell-free virus.

### 3.2. Endogenous MLVs

At least 100 times over the course of evolution, MLVs have infected cells of the mouse germline. Once the viral DNA has integrated into the germline DNA, it is passed from parents to offspring just like any other mouse gene. The biology of these “endogenous” MLVs and their effects on their hosts are quite complex and are considered in other articles in this series.

### 3.3. Resistance to MLV

While MLVs are generally benign at the cellular level, they do induce both lymphomas and neurological diseases in mice. Mice have evolved a number of resistance mechanisms that inhibit the growth of MLVs; MLVs have, in turn, developed strategies for evading these defense mechanisms. 

#### 3.3.1. Superinfection Interference

Two genes inducing strong resistance to specific envelope classes of MLV have been described: Fv-4 and Rmcf [102, 103]. Both of these genes have been found to function by superinfection interference: in other words, the genes encode glycoproteins which bind MLV receptors, rendering the receptors unavailable for incoming viruses. Fv-4 blocks the ecotropic receptor, mCAT1, whereas Rmcf blocks the MCF receptor XPR1. It seems reasonable to imagine that these genes were originally introduced into the mouse genome as the Env genes of endogenous MLVs.

#### 3.3.2. Fv1 Restriction

Fv1 restriction was the first system for resistance to MLV to be described in mice [104]. Inbred mouse strains carry the “n” allele, the “b” allele, or the “nr” allele at the Fv1 locus. In turn, naturally occurring MLVs may be N-tropic or B-tropic. Fv1^n^ or Fv1^nr^ mice are partially resistant to B-tropic MLVs, while the Fv1^b^ locus encodes partial resistance to N-tropic MLVs (Fv1^nr^ mice are resistant to some N-tropic MLVs as well as B-tropic MLVs). Passage of an MLV in the restrictive host may ultimately lead to the selection of a viral variant that has lost its sensitivity to Fv1 restriction; these laboratory isolates, such as Moloney MLV, are termed NB-tropic. XMRV is unique in that it is restricted by both Fv1^n^ and Fv1^b^ [105].

Despite many years of investigation, the mechanism of Fv1 restriction is still not well understood. The Fv1 gene product seems to be a somewhat degenerate retroviral Gag protein [106]. Genetic data indicate that it binds to a specific site in the N-terminal domain of CA in the mature core of the incoming virus particle. This interaction blocks infection at a point between reverse transcription and integration of the viral DNA. The Fv1 protein is present in cells at extremely low levels [107]; in fact, restriction can be blocked or “abrogated” by infection with a single particle of the restricted type [108]. Particles which have been inactivated by heat or gamma irradiation can retain the ability to abrogate Fv1 restriction [109]. 

Biochemical analysis of the Fv1 restriction machinery has proven extremely difficult, but it appears that the ability of the Fv1 protein to multimerize [110] is an essential element in restriction [111]. The specific binding of the protein to CA protein of the restricted type seems to occur only when the mature CA is in a lattice, as in the viral core; this binding was recently demonstrated, for the first time, using CA protein arrayed on lipid nanotubes [112]. 

While the Fv1 restriction system is, as far as is known, found only in mouse cells, human cells possess a somewhat analogous restriction system effected by the TRIM5*α* protein. TRIM5*α* was discovered by virtue of its ability to restrict HIV-1, but it is also active against some MLVs; remarkably, like the Fv1 gene product, it distinguishes between N-tropic and B-tropic MLVs [113].

#### 3.3.3. APOBEC3 Restriction

All placental mammals have at least one member of the APOBEC3 gene family; humans and chimpanzees have seven APOBEC3 genes [114, 115]. APOBEC3 proteins can be incorporated into retrovirus particles, and they interfere with viral replication during reverse transcription when the APOBEC3-bearing virus particle infects a new host cell. APOBEC3s are cytidine deaminases with one or two zinc-coordinating motifs that are instrumental in the restriction of viral replication. It seems likely that the primary function of APOBEC3s is protection of the mammalian host against pathogens (or intracellular parasites such as retrotransposons): mice lacking mouse APOBEC3 (mA3) survive and reproduce normally but are very sensitive to retrovirus infection [116, 117].

One way in which APOBEC3 proteins inactivate retroviruses is by hypermutation. By deaminating deoxycytidine to deoxyuridine in minus-strand DNA during the synthesis of viral DNA, they bring about a G to A change in the plus-strand. Many susceptible viruses have been shown to incur very high levels of G to A mutation as a result of APOBEC3 action. However, it is now clear that APOBEC3 proteins act on retroviruses in other ways as well. For example, the degree of inactivation of HIV-1 by human APOBEC3G (hA3G) does not necessarily correlate with the level of G to A mutation (reviewed in [118]), and hA3G has been shown to affect both the synthesis and integration of HIV-1 viral DNA [119].

There are two isoforms of mA3, containing or lacking exon 5. Most studies on mA3 have used the form lacking the exon. MLVs show dramatic differences in their sensitivity to this mA3: both XMRV and AKV (the endogenous ecotropic MLV in AKR mice, a mouse line bred for high leukemia incidence) are far more sensitive to inactivation by mA3 than Moloney MLV (which was selected for rapid growth and leukemogenicity by passage in mice over a period of years) [105, 120–122]. Moreover, when DNA of XMRV or AKV is synthesized in the presence of mA3, it contains large numbers of G to A mutations [120, 121], but these mutations are not detectably induced in Moloney MLV by mA3 [100, 123]. Presumably, the creation of the AKR mouse strain entailed the selection of mice that provide a maximally permissive environment for AKV, and thus, this virus has not faced selective pressure leading to mA3 resistance. In contrast, selection during passage of Moloney MLV has led to partial resistance to inactivation by mA3, and apparently complete resistance to the hypermutational effects of mA3. The mechanisms underlying these resistance phenomena are unknown. It should be noted that in HIV-1, one of the “accessory proteins”, that is, Vif, is responsible for viral resistance to hA3G. Vif functions by binding to hA3G and inducing its proteasomal degradation. However, as emphasized above, MLVs do not encode accessory proteins, and the resistance of Moloney MLV to mA3 must reside in its Gag, Pol, and/or Env protein. As mA3 is packaged efficiently in Moloney MLV particles [100, 123], the resistance does not depend upon exclusion of mA3 from the virus. 

The biology of MLV restriction by the mA3 containing exon 5 is somewhat different from the foregoing: mA3 protein containing this exon can be cleaved by MLV PR, leading to the inactivation of this mA3 within MLV particles [124].

#### 3.3.4. Restriction by Tetherin

**Figure 7:**
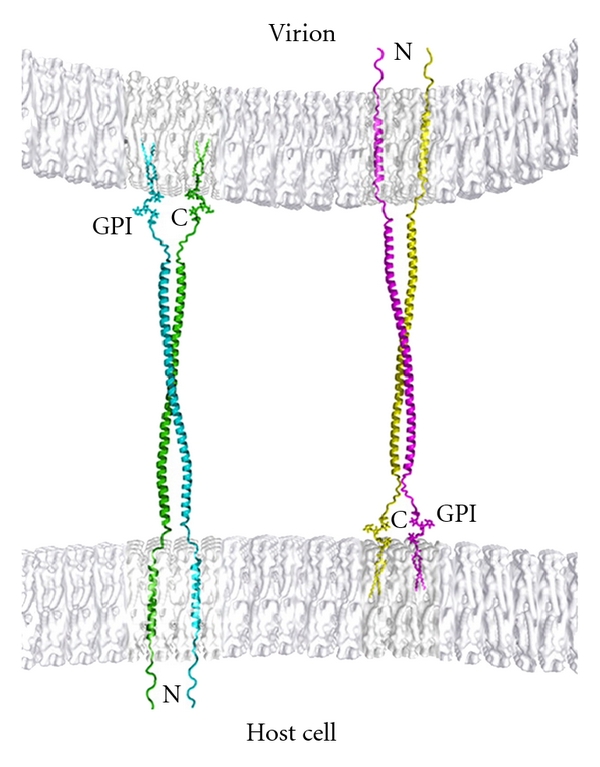
Hypothetical mechanism of restriction by tetherin. The cellular restriction factor tetherin can act as a bridge between two membranes. Tetherin contains a transmembrane domain at its N-terminus and is anchored to a membrane by a glycophosphatidyl linkage at its C-terminus. It also dimerizes due to a parallel coiled-coil structure between the termini of the protein. Anchorage to membranes at both ends apparently enables tetherin to “trap” virus particles, preventing their escape from the virus-producing cell. It is not known which end of the protein is embedded in the cellular membrane and which in the viral membrane. (Figure reproduced with permission from [22].)

Recently, yet another antiviral restriction system has been discovered, mediated by the host protein “tetherin” (also known as CD317, BST2, or HM1.24) [125]. Tetherin is a membrane protein with a very unusual topology: it has a cytoplasmic N-terminus, followed by a transmembrane helix, an extended ectodomain, and a C-terminus associated with the plasma membrane by a glycophosphatidyl inositol linkage. Tetherin dimerizes via the ectodomain, which forms a coiled coil ~90 Å long. The presence of membrane anchors at both ends of the molecule evidently gives it the ability to physically link released virus particles to the surface of the virus-producing cell, effectively preventing their escape into the surrounding medium (see Figure 7) [22].

Tetherins inhibit the release of all retroviruses tested, and also of filoviruses such as Ebola, arenaviruses such as Lassa, and herpesviruses such as Kaposi's sarcoma-associated herpesvirus. They are constitutively expressed on some cell surfaces and are inducible by type I interferon in others. Mouse tetherin has been shown to inhibit the replication of MLV [126]. While lentiviruses have several alternative countermeasures against tetherins, including the HIV-1 accessory protein Vpu (reviewed in [127]), no resistance mechanisms in MLVs have yet been described.

## 4. Concluding Remarks

It is clear that MLVs have provided an extraordinary wealth of information about retroviruses, both as physical objects and as living organisms. They (and other gammaretroviruses, such as gibbon ape leukemia virus) are now being developed as vectors for gene therapy. As has been indicated throughout this paper, the contrasts with other retroviruses such as HIV-1 help to illustrate the range of possibilities by which viruses solve common problems. Finally, as with all viruses, MLVs provide a window into the “black box”, an unparalleled opportunity to learn about the cells and organisms that they infect. Indeed, many cellular proteins have been shown to participate in MLV replication; while this large topic is beyond the scope of this paper, it is the focus of a fascinating review by Goff [128].

## Figures and Tables

**Table 1:** MLV receptors on NIH/3T3 mouse cells.

Virus class	Example	Receptor	Reference
Ecotropic	Moloney	mCAT1	[12]
	MLV		
Polytropic	MCF247	XPR1	[13–15]
Amphotropic	1504A	SLC20A2	[16, 17]
		SLC20A1	
10A1	10A1	or	[16, 17]
		SLC20A2	

The table lists the receptors for MLVs found on NIH/3T3 mouse cells. The diversity of MLV receptors is discussed in more detail in other articles of this series.
